# The duality of temporal encoding – the intrinsic and extrinsic representation of time

**DOI:** 10.3389/fpsyg.2015.01288

**Published:** 2015-08-31

**Authors:** Ronen Golan, Dan Zakay

**Affiliations:** ^1^School of Psychological Sciences, Tel Aviv UniversityTel Aviv, Israel; ^2^Psychology Department, Interdisciplinary Center HerzliyaHerzliya, Israel

**Keywords:** time perception, temporal encoding, time representation, FFA, PPA, Cerebellum, Caudate, Thalamus

## Abstract

While time is well acknowledged for having a fundamental part in our perception, questions on how it is represented are still matters of great debate. One of the main issues in question is whether time is represented intrinsically at the neural level, or is it represented within dedicated brain regions. We used an fMRI block design to test if we can impose covert encoding of temporal features of faces and natural scenes stimuli within category selective neural populations by exposing subjects to four types of temporal variance, ranging from 0% up to 50% variance. We found a gradual increase in neural activation associated with the gradual increase in temporal variance within category selective areas. A second level analysis showed the same pattern of activations within known brain regions associated with time representation, such as the Cerebellum, the Caudate, and the Thalamus. We concluded that temporal features are integral to perception and are simultaneously represented within category selective regions and globally within dedicated regions. Our second conclusion, drown from our covert procedure, is that time encoding, at its basic level, is an automated process that does not require attention allocated toward the temporal features nor does it require dedicated resources.

## Introduction

Encoding temporal information of our surrounding is a fundamental cognitive and neural process. A faithful representation of sensory information includes not just the WHAT and WHERE, but also the temporal characteristics of a stimulus or an event, i.e., the WHEN; however, we do not possess any temporal sensor. Moreover, opposed to other sensory information that can be turned on and off, temporal experience seems to be continuous and thus makes the task of tracing its nature much more challenging. Our goal in this study was to try and overcome this challenge and establish a methodology that can help us monitor, at the neural level, the automatic nature of temporal encoding.

While cognitive internal clocks models capture quite accurately our overt prospective time experience (Treisman, 1963; Church, 1984; Zakay, 1989; Zakay and Block, 1996), they still lack the mechanisms underlying our covert continuous temporal encoding and their relations to our conscious psychological time experience. Gaining a better understanding on the way temporal information is processed from its initial encoding stage up to its psychological experience stage along with the neural substrates underlying it, may assist us in establishing a more comprehensive model of temporal processing.

Neural models of temporal encoding can be broadly divided into two categories – intrinsic vs. extrinsic or dedicated models. Intrinsic models relay on the idea that time is inherent to neural dynamics (i.e., oscillations, rhythmical or state-dependent models) and is represented locally in the brain. In this category we can find single cell models (Leon and Shadlen, 2003), where time is represented by the actual activity of a specific neuron: either excitatory (i.e., more is more; higher activity means longer duration; Pariyadath and Eagleman, 2007; Eagleman, 2008), or inhibitory (Constantinidis et al., 2002) by inhibiting a response for a specified duration, as well as “State Dependent Models” where time is represented based on the general properties of a specific neural network (Buonomano and Merzenich, 1995; Buonomano, 2000; Karmarkar and Buonomano, 2007). Extrinsic or dedicated models focus on central specialized time keeping mechanism, such as central internal clock models (Meck, 1996, 2005; Matell and Meck, 2000; Hofstötter et al., 2002; Mauk and Buonomano, 2004) where time is globally represented based on an oscillating unit; or, network models involving several centers in the brain such as the Cerebellum-SMA-Basal Ganglia circuit (Ferrandez et al., 2003) along with scattered representation of time, where several internal clocks represent time for different modalities (Goldstone and Lhamon, 1974; Lhamon and Goldstone, 1974; N’Diaye et al., 2004).

Naturally there are pros and cons for each of these approaches. As expressed by Ivry and Schlerf (2008), dedicated models have difficulties in accounting for impaired encoding of time as a result of modulations in neural activity. At the same time intrinsic models suffer from poor explanatory power when accounting for cross modality effects on time perception (Warm et al., 1975; Roberts, 1982). Moreover, intrinsic models will have difficulties to account for global effects on time perception, like attention, while extrinsic models will have difficulties explaining local representation of time of specific types of stimuli in category selective areas.

Either intrinsic or extrinsic, current models seem to lack a more radical approach, namely that temporal characteristics are integral to stimulus processing and should be represented within category or feature selective neural populations.

A related issue in the study of time perception and temporal representation is whether time encoding and perception are automated, continuous, pre-attentive processes or do they require the allocation of attention or dedicated resources to a well-defined duration or interval; in other words, the overt vs. the covert encoding of time. Some studies suggested that implicit timing has distinct mechanisms (Coull and Nobre, 2008) while other studies (Praamstra et al., 2006) suggested that implicit and explicit timing relies on common mechanisms. Nevertheless, the mere fact that time can be represented implicitly (regardless of the mechanisms underlying it) suggest that time encoding has an automatic pre-attentive characteristic that should be addressed.

In the current study, our main challenge was to establish a method for tracking covert temporal processing at the neural level. Based on the assumption that time is an integral part of perception (Zakay et al., 2014), we expected temporal encoding to be represented within sensory modules, associated with the representation of the stimulus, rather than solely associated with either mere neural dynamics or distinct generic regions. More specifically, we expected category selective areas that typically encode information about the shape of visual stimuli to also encode its temporal information. Consequently, our two main goals in this study were to test whether temporal representation is an integral part of the stimulus representation occurring within category selective brain regions that elicit a selective response to specific stimuli; and to test if the temporal encoding is an automatic process occurring without allocating attention toward the temporal characteristics of the stimulus and without involving any motor reaction or motor planning.

In order to achieve these goals, functional MRI was used to investigate both the continuous property of time, namely the encoding of time without allocating dedicated resources for its processing; and the dual simultaneous representation of time, both intrinsically and extrinsically. We based our method on the novelty-habituation effect of neural population behavior. Perceiving neurons are excited when presented with a novel stimuli; a repetitive presentation of a stimulus will instigate an habituation process which is associated with neuronal inhibitory processes. The presentation of a novel stimulus will reinstate neuronal excitation (i.e., dishabituation). The processes described above suggest some sort of a feature comparison mechanism as per novelty detection. Indeed, Sokolov (1990) suggested a feature neural comparison model which was supported by several studies (see Siddle, 1991; Ben-Shakhar et al., 2000; Ben-Shakhar and Gati, 2003). Feature mismatch during this comparison procedure is assumed to be the basis for neuronal excitation.

A step toward adopting this novelty/habituation approach in fMRI was practiced by Grill-Spector et al. (1999) using a method called fMR-Adaptation (fMR-A; for an overview see Grill-Spector and Malach, 2001 and Grill-Spector et al., 2006). Grill-Spector and Malach (2001) repeatedly exposed subjects to visual objects stimuli in order to study invariant object properties (e.g., rotation, illumination) in high-order object areas [Lateral Occipital Complex (LOC)]. These studies demonstrated that by examining which object properties showed release from adaptation (i.e., dishabituation) the nature of representation of different object features in LOC (e.g., invariance to object rotation) would surface.

We believe that fMR-A can be applied to the study of duration encoding. Specifically, we aimed to apply the fMR-A technique in order to find brain regions that show dishabituation to temporal information of visual stimuli. We estimated that such a procedure could be used to localize brain areas that are sensitive to temporal information and therefore are involved in the encoding stages of time perception. Consequently, we designed a covert procedure where subjects were not informed of the temporal settings of the experiment, while engaged in a non-motor, non-temporal task. Moreover, we selected distinct brain regions that typically encode information about the shape of visual stimuli and asked if temporal encoding is performed within these regions. More specifically, we functionally localized the face-selective areas in the inferior occipital lobe (occipital face area – OFA) and in the fusiform gyrus (fusiform face area – FFA) as well as scene-selective areas such as the parahippocampal place area (PPA) and the transverse occipital sulcus (TOS). The reason for selecting these regions is that they are well-defined and easy to functionally localize.

In line with the neural behavior where repetitive exposure to the same feature or stimulus decreases the neural activation of the subpopulation representing it (Grill-Spector et al., 2006; Krekelberg et al., 2006) we expect that neural activation of subpopulations specializing in representing a specific stimulus will be positively correlated to the variance in stimulus features, including its temporal features. That is to say, the higher the variance between repetitive stimuli – the higher the activation of the neural subpopulation would be. Thus, we hypothesized that if the specific regions mentioned above (FFA, OFA, PPA, and TOS) encode the temporal features of their category stimuli, they should be sensitive to variance in the exposure duration of these stimuli. More specifically, we expected that the higher the variance in exposure duration – the larger the elicited activation of the specific region should be.

## Materials and Methods

### Participants

Fifteen healthy volunteers (12 females and 3 males; mean age = 28.47 years, SD = 4.37) participated in the Experiment. All subjects were either undergraduate students or post graduates with advanced degrees (i.e., MAs, PhDs, MD). All subjects gave informed consent for participation in the study, which was approved by the ethics committee of the Tel-Aviv Sourasky Medical Center.

### Stimuli

All visual stimuli were grayscale. Functional localizer images were miscellaneous 300 pixels × 300 pixels photographs of 80 different faces, 80 different types of objects (e.g., ball, apple, barrel) and 80 different types of natural scenes (e.g., houses, landscapes).

**FIGURE 1:**
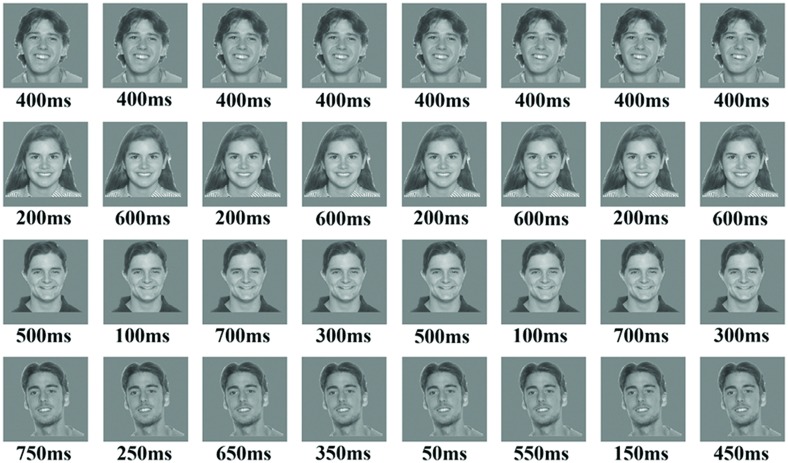
**The figure presents an example of the four blocks in the temporal condition using face stimuli**. The upper row is an example of the baseline block with 0% variance, while the fourth row is an example of block 4 with 50% variance in durations. The parameters for each block consisted of the following: (a) Number of stimuli in a block was 16; (b) ISI was 350 ms; (c) block duration was 12-s; (d) total exposure to stimulus in all blocks was 6400 ms.

Stimuli used in the temporal condition scans were four different photographs of faces (**Figure 1**) and four different photographs of houses with an average intensity of 160. Background was grayscale with an intensity of 160 to match the average intensity of the stimuli. Faces and houses images were randomly selected from the images used in the localizer scans; all faces images had the same expression. An altering colored fixation point of 8 pixels × 8 pixels was presented in the center of the images, using Matlab 7 (Psychtoolbox, Brainard, 1997).

Stimuli were presented with Matlab 7 (Psychtoolbox, Brainard, 1997) and were projected onto a screen located at the back of the scanner through a projector. Subjects viewed the stimuli through a mirror that was placed on the upper part of an eight channel head coil in front of their eyes.

### Procedure

#### Functional Localizer Scans

A block-design functional localizer was used to identify regions of interest (ROI) of two categories, Faces and Scenes, using three stimulus classes: Faces, Scenes, and Objects. Functional localizer scans consisted of four blocks (16-s each) of each stimuli category (each condition repeated four times in each scan) and five blocks (16-s each) of a baseline fixation point. For each block, 20 images form a single stimulus class were presented (300-ms per image, with 500-ms interstimulus interval); with an addition of a 12-s dummy block, each localizer scan lasted for 4-min and 44-s. In order to ensure general vigilance, subjects were instructed to memorize all consecutive identical images (1-back task). At the end of each scan subjects were asked to report their findings.

#### Temporal Conditions Scans

For the temporal scans we used a block-design, where each scan consisted of four condition blocks (12-s each) presented twice (total of eight blocks), and nine baseline fixation blocks (12-s each). In each block subjects were exposed to 16 repetitions of the exact same stimulus of the same category (either a face or a house). For each condition block, stimulus duration had a distinct degree of variance (i.e., 0, 12.5, 25, and 50%), while the ISI between stimuli and the total exposure to a stimulus within a block remained constant across all blocks. In order to make sure that the effect is not duration dependent, we used two sets of durations, as well as two types of ordering (i.e., the first duration in a block could be either the relatively long duration or the relatively short one). Thus, eight subjects were exposed to the first set, and seven subjects to the second set.

In the 0% variance condition, each repetition had the same duration (either 400-ms for some subjects; or 500-ms for others). In the 12.5% variance condition we used two types of durations (either 600 and 200-ms with eight repetitions each; or 200 and 800-ms; see **Figure 1**). For the 25% variance, we used four types of durations with four repetitions each (either 100, 300, 500, and 700-ms; or 150, 300, 700, and 850-ms). And finally, for the 50% variance condition we used eight types of durations with two repetitions each (either 50, 150, 250, and 350-ms, 450, 550, 650, and 750-ms; or 100, 250, 400, and 450-ms, 550, 600, 750, and 900-ms). Within the first set of durations ISI was 350-ms and total exposure time within a block was 6400-ms for all conditions. Within the second set of durations ISI was 250-ms and the total exposure time within all conditions was 8000-ms.

In total there were four scans using faces images and four scans using houses images. Conditions were counterbalanced both for order and images so that a specific image will appear across all types of conditions.

A colored fixation dot was presented over each image. Participants were instructed to detect a distinct pattern of the colored fixation point (e.g., a sequence of two consecutive blue dots). To avoid any motor reaction during the experiment, at the end of each scan subjects were instructed to report in which of the images the distinct sequence appeared more frequently.

### MRI

#### Dada Acquisition

MRI data was collected in a 3T GE MRI scanner. Echo planar imaging sequence was used to collect fMRI data with the following parameters: TR = 2-s, TE = 35-ms, flip angle: 90°, 34 slices per TR, slice thickness: 4 mm no gap, matrix 64 × 64, and FOV 256-mm.

#### Data Analysis

fMRI data analysis was conducted using statistical parametric mapping (SPM2^1^). Images acquired during the first 12-s of each scan were discarded. Preprocessing of EPI images included slice timing correction, realignment, normalization to a standard template [Montreal Neurological Institute (MNI), voxel size 3 × 3 × 3], and spatial smoothing with an 5 mm × 5 mm × 5 mm full-width at half-maximum (FWHM) Gaussian kernel.

#### Functional Localizer Data Analysis

We used data from the localizer scans to identify regions dedicated for processing of faces and scenes for each subject independently. The FFA and OFA for face selective area were defined by using a Faces > Objects contrast. For the scenes selective area a Scene > Object contrast was used to define the PPA and the TOS.

#### Temporal Conditions Scans within Category Selective Areas Data Analysis

A general linear model was estimated for each individual subject using SPM2, Finite Impulse Responses (FIR) and Fitted event time courses were extracted from the predefined ROIs using the MarsBar Toolbox for SPM2^2^ and imported into MATLAB R2010a and SPSS21 for statistical analysis. For the statistical analysis we used the maximum value of the FIR signal percent change from each time course and the two highest values surrounding it (a total of 6-s out of a 12-s block).

#### Whole Brain Second Level Analysis for Temporal Conditions Scans

A general linear model was estimated for each individual subject. A whole brain analysis was performed for each subject using a parametric contrast (i.e., -3, -1, 1, 3 for the temporal conditions, respectively), while disregarding the distinction between faces stimuli and houses stimuli. As a result, for the second level analysis each condition included data from 16 blocks rather than 8 for the category selective areas.

Contrasts from each subject were used for a second level analysis (i.e., “basic models” *t*-Test in SPM2). Based on the second level whole brain analysis we extracted common ROIs of significant activations (*p* = 0.0001 uncorrected). FIR and Fitted event Time courses were extracted from each individual subject, based on extracted ROIs using the MarsBar Toolbox for SPM^2^ and imported into MATLAB R2010a and SPSS21 for statistical analysis. For the statistical analysis we used the maximum value of the FIR signal percent change from each time course and the two highest values surrounding it (a total of 6-s out of a 12-s block).

## Results

### Face/Scene Localizer

**FIGURE 2:**
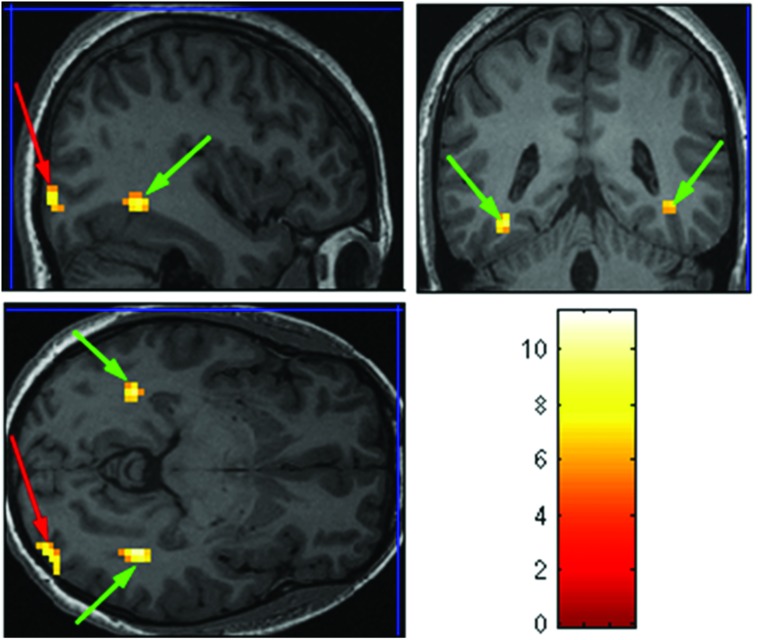
**Extracted regions of interests (ROIs) based on the Face-Scene-Object functional localizer scans as seen in one of the subjects**. Green arrows pointing on the fusiform face area (FFA); red arrows pointing on the occipital face area (OFA).

**FIGURE 3:**
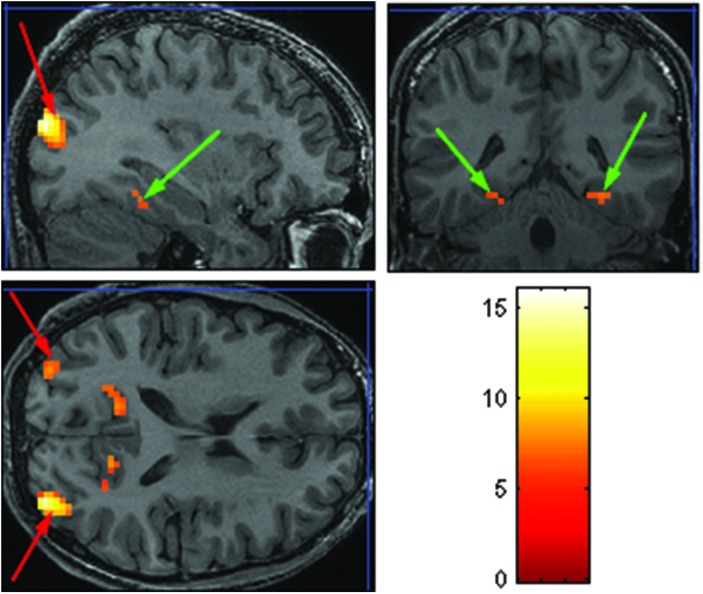
**Extracted ROIs based on the Face-Scene-Object functional localizer scans, as seen in one of the subjects**. Green arrows pointing on the parahippocampal place area (PPA); red arrows pointing on the transverse occipital sulcus (TOS).

The purpose of the Face-Scene localizer was to extract specific ROIs and to test how these ROIs respond to the temporal manipulation. We used the Faces > Objects contrast (*p* = 0.001 uncorrected) to identify the FFA (**Figure 2** green arrows) and OFA (**Figure 2** red arrows) and the Scene > Object contrast (*p* = 0.001 uncorrected) to identify the PPA (**Figure 3** green arrow) and TOS (**Figure 3** red arrow). Based on our findings and on previous studies (Kanwisher et al., 1997; Loﬄer et al., 2005; Rotshtein et al., 2005), we focused our analysis on activations in the right hemisphere (i.e., rFFA, rOFA, rPPA, and rTOS), which elicited stronger activations than in the left hemisphere (for example only 6 out of the 15 Ss showed left OFA activation).

Out of our 15 subjects one subject did not show any specific activations to neither faces nor houses stimuli. Moreover, one subject did not show activations only in rOFA, another subject had no activations only in rPPA and a fourth subject had no activations in rTOS. Thus for the analysis of temporal conditions within category selective ROIs we used data from 14 Ss for rFFA and from 13 Ss for rOFA, rPPA, and rTOS. However, for the second level analysis where no pre-defined ROIs where required, data from all participants was included.

### Temporal Conditions within Category Selective Areas

After identifying face and scene selective areas for each subject (e.g., rFFA, rOFA, rPPA, and rTOS), we extracted the time courses for each temporal condition from each ROI for each subject.

**FIGURE 4:**
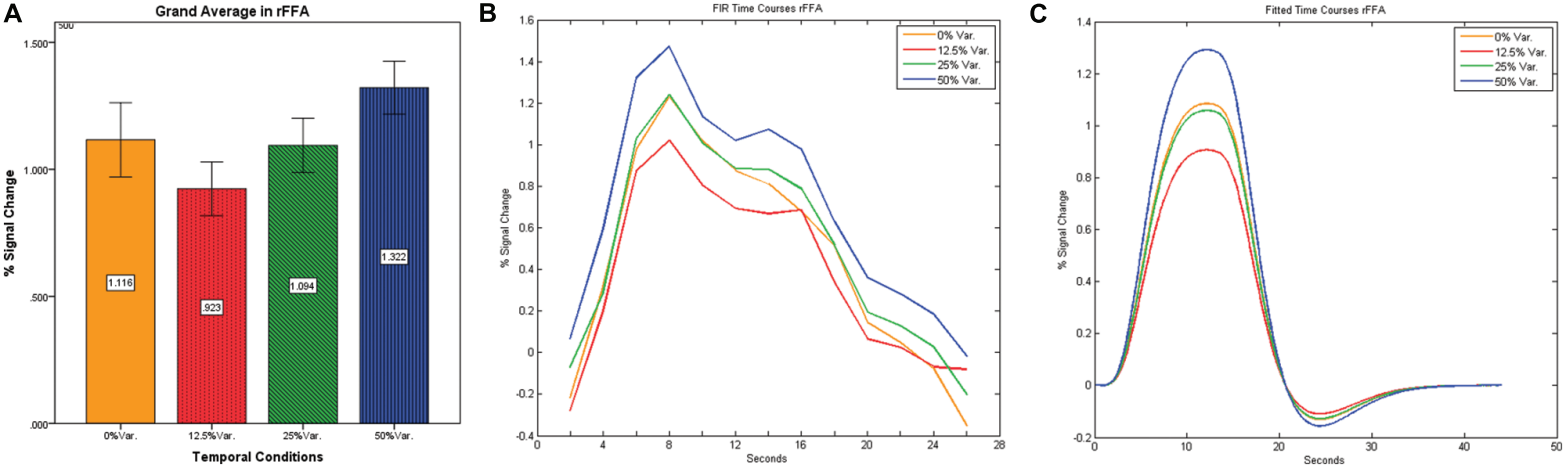
**(A)** Grand average of the mean percent signal change in rFFA for faces stimuli for all four conditions, showing a gradual increase in activation between conditions having variance in durations (i.e., 12.5, 25, and 50% variance) while the first condition with 0% variance showing a greater activation than the 12.5 and 25% variance conditions. **(B)** Grand average of FIR event time courses extracted from the rFFA; **(C)** Grand average of Fitted event time courses extracted from the rFFA.

**FIGURE 5:**
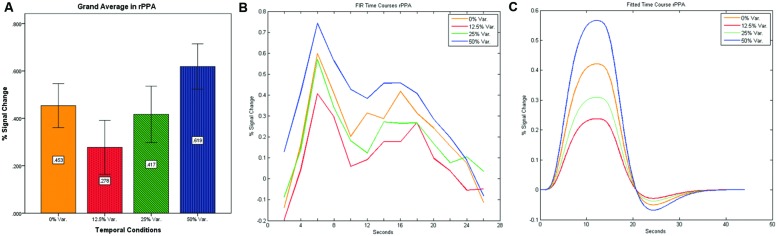
**(A)** Grand average of the mean percent signal change in rPPA for faces stimuli for all four conditions, showing a gradual increase in activation between conditions having variance in durations (i.e., 12.5, 25, and 50% variance) while the first condition with 0% variance showing a greater activation than the 12.5 and 25% variance conditions. **(B)** Grand average of FIR event time courses extracted from the rPPA; **(C)** Grand average of Fitted event time courses extracted from the rPPA.

As can be seen in **Figures 4** and **5** we found that the 0% variance condition yielded a lower activation than the 50% variance condition both in rFFA [*t*(82) = -2.29, *p* = 0.025] and rPPA [*t*(76) = -2.48, *p* = 0.015]. Moreover, a gradual increase in mean activations appeared between the second, third, and fourth conditions in both the rFFA and the rPPA. The first condition yielded a higher activation than the second condition in both ROIs. A one-way ANOVA test over all four conditions revealed a significant main effect of the difference in mean activations for the rFFA with faces stimuli [*F*(3,164) = 7.77, *p* < 0.00007] and for the rPPA with houses stimuli [*F*(3,152) = 7.01, *p* = 0.0002]. A linear trend test using contrast coefficients of [-3, -1, 1, 3] yielded a significant effect in both rFFA [*t*(164) = 3.01, *p* = 0.003] and rPPA [*t*(152) = 2.68, *p* = 0.008].

**FIGURE 6:**
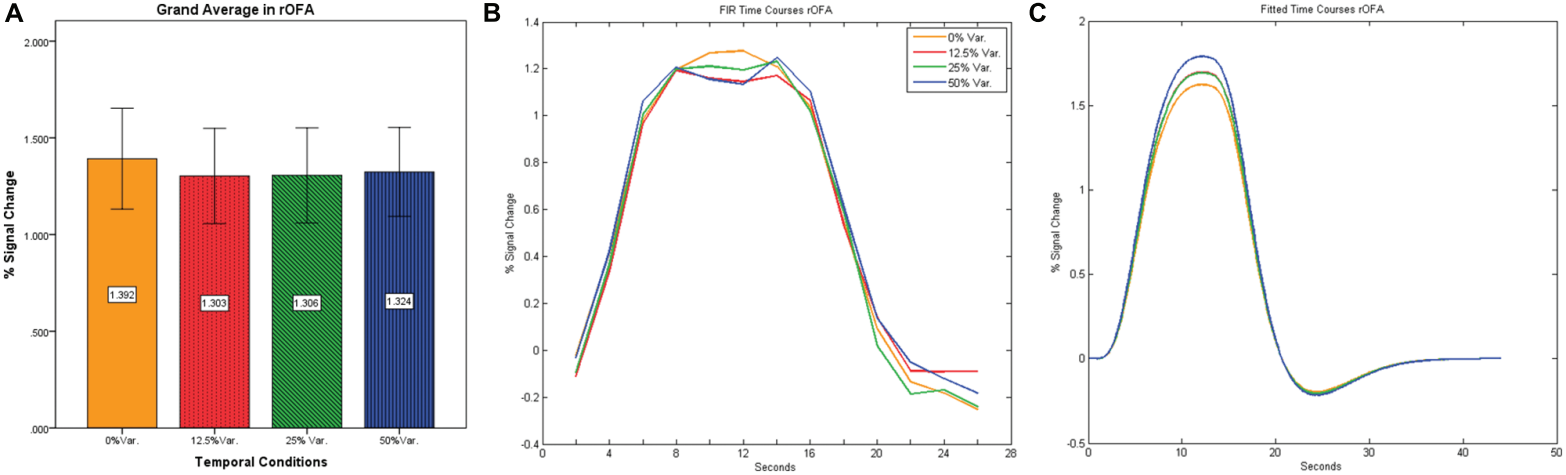
**(A)** Grand average of the mean percent signal change in rOFA for faces stimuli for all four conditions, showing no sensitivity to temporal manipulation. **(B)** Grand average of FIR event time courses extracted from the rOFA; **(C)** Grand average of Fitted event time courses extracted from the rOFA.

**FIGURE 7:**
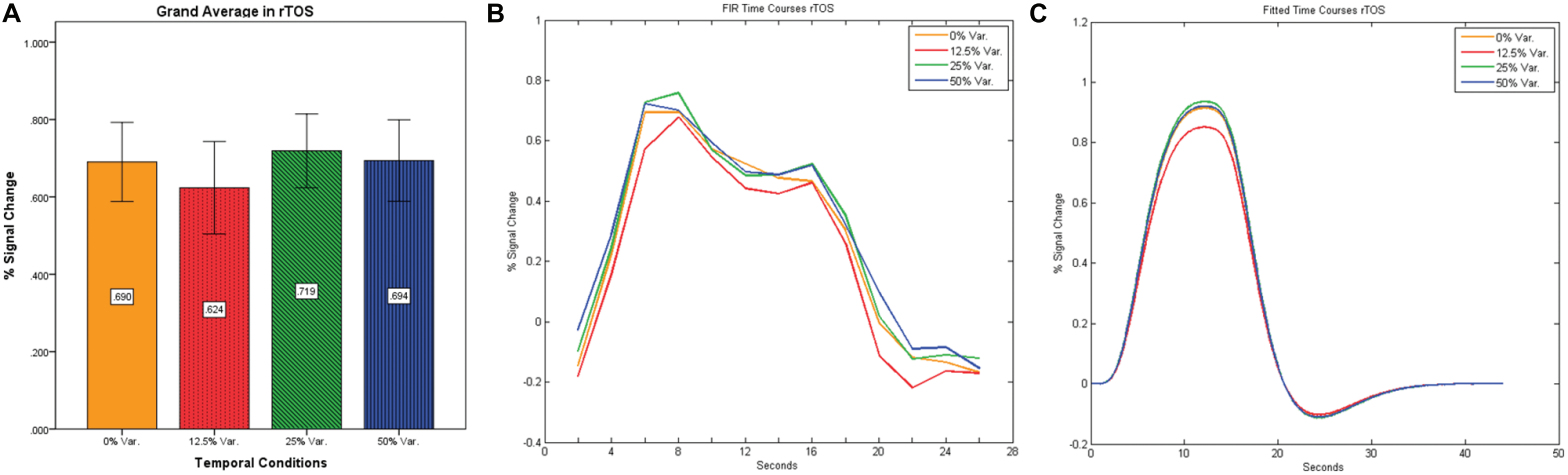
**(A)** Grand average of the mean percent signal change in rTOS for faces stimuli for all four conditions, showing no sensitivity to temporal manipulation. **(B)** Grand average of FIR event time courses extracted from the rTOS; **(C)** Grand average of Fitted event time courses extracted from the rTOS.

In contrast to the rFFA and rPPA, no effect was found in rOFA [*F*(3,152) = 0.114, *p* = 0.952] nor in rTOS [*F*(3,152) = 0.595, *p* = 0.62] (see **Figures 6** and **7**). Consequently no significant linear trend was found within these ROIs, i.e., rOFA – [*t*(152) = -0.37, *p* = 0.714]; rTOS – [*t*(152) = 0.45, *p* = 0.653].

### Second Level Analysis of the Temporal Conditions – Whole Brain Analysis

**FIGURE 8:**
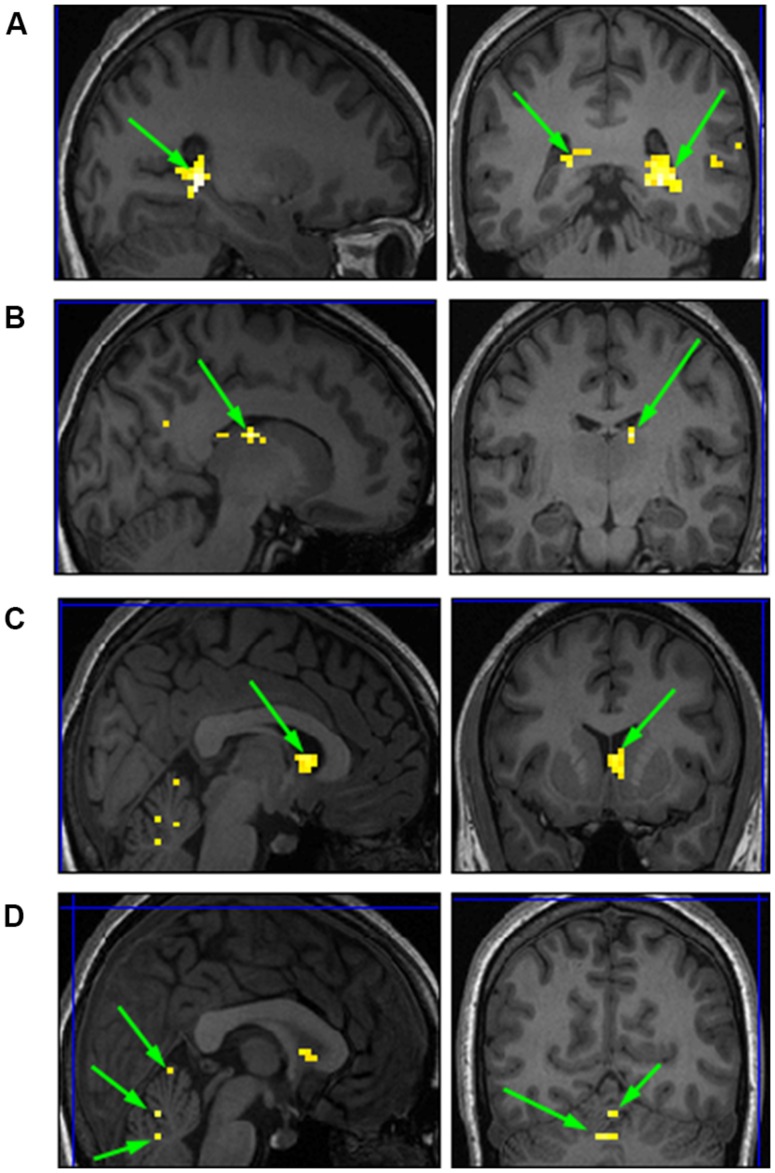
**Common ROIs extracted based on a second level analysis. (A)** right and left Parahippocampal Gyrus; **(B)** right Thalamus; **(C)** right Caudate; **(D)** right and left Cerebellum.

We conducted a second level analysis including all 15 subjects while disregarding stimulus type (i.e., faces or houses) and addressing only the four temporal conditions differing in variance of the duration exposure of the stimulus to extract common ROIs sensitive to the temporal variance manipulation. Analysis yielded four bi-lateral distinct ROIs. MNI coordinates were identified based on Talairach human brain mapping, returning the following four regions (**Figure 8**): (1) Right and Left Parahippocampal Gyrus and; (2) the right and left Thalamus; (3) right and left Caudate; and (4) right and left Cerebellum with specificity to the Pyramis, Inferior Semilunar lobule, and Culmen. (see **Table 1** for details of ROIs and MNI Coordinates).

**Table 1:** Common regions of interests (ROIs) based on a second level analysis.

Montreal Neurological Institute coordinates (x, y, z)	Talairach mapping
(27, -42, -6)	Right Parahippocampal Gyrus
(-18, -45, 3)	Left Parahippocampal Gyrus
(3, 9, 6)	Right Caudate Body/Head
(-3, 15, 6)	Left Caudate Body/Head
(24, -33, 18)	Right Caudate Tail
(-6, 0, 12)	Left Caudate Body
(12, -15, 18)	Right Thalamus
(-9, -24, 12)	Left Thalamus – Pulvinar
(0, -63, -24)	Left/Right Cerebellum – Pyramis
(-3, -63, -36)	Left Cerebellum – Inferior Semi-Lunar Lobule
(-3, -57, -3)	Left Cerebellum – Culmen

**FIGURE 9:**
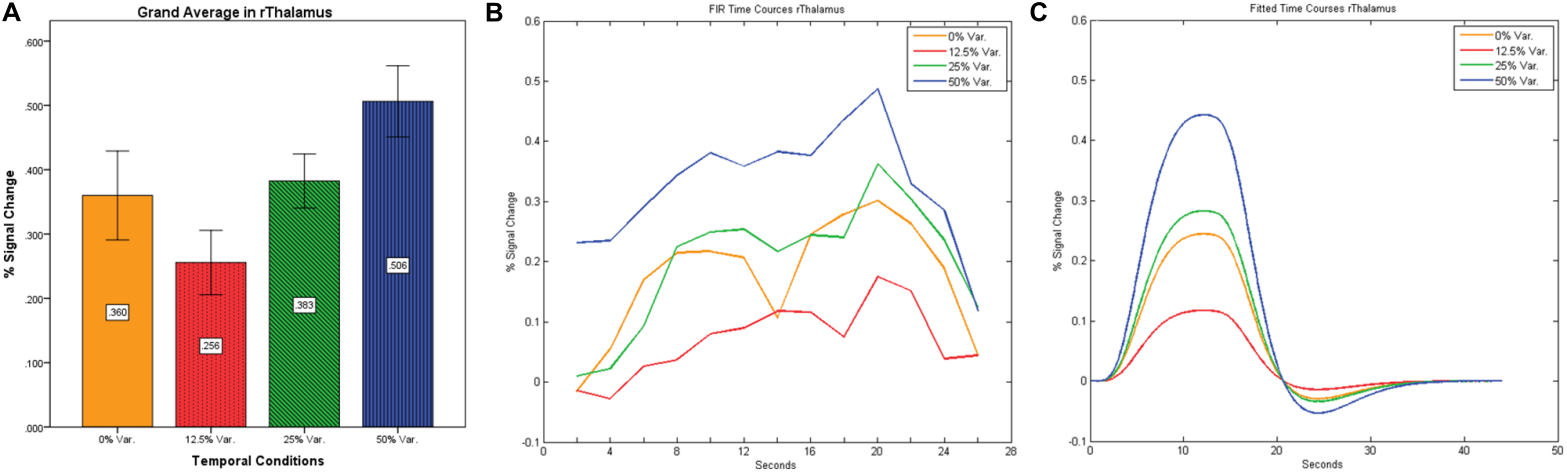
**(A)** Grand average of the mean percent signal change in rThalamus, showing a gradual increase in activation between conditions having variance in durations (i.e., 12.5, 25, and 50% variance) while the first condition with 0% variance showing a greater activation than the 12.5% variance conditions. **(B)** Grand average of FIR event time courses extracted from the rThalamus (12, -15, 18). FIR time courses peak at about 20-s after block onset; **(C)** Grand average of Fitted event time courses extracted from the rThalamus.

**FIGURE 10:**
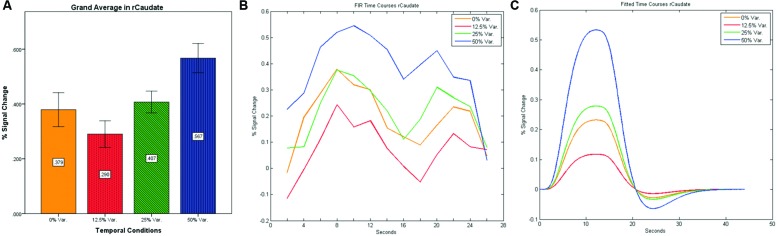
**(A)** Grand average of the mean percent signal change in rCaudate for all four conditions, showing a gradual increase in activation between conditions having variance in durations (i.e., 12.5, 25, and 50% variance) while the first condition with 0% variance showing a greater activation than the 12.5% variance conditions. **(B)** Grand average of FIR event time courses extracted from the rCaudate (3, 9, 6). FIR event time courses peak at around 10-s after block onset; **(C)** Grand average of Fitted event time courses extracted from the rCaudate.

**FIGURE 11:**
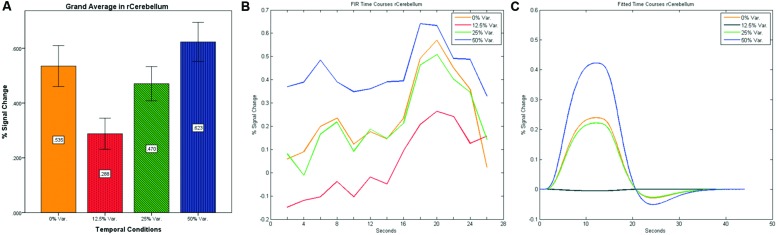
**(A)** Grand average of the mean percent signal change in r/lCerebelum for all four conditions, showing a gradual increase in activation between conditions having variance in durations (i.e., 12.5, 25, and 50% variance) while the first condition with 0% variance showing a greater activation than the 12.5 and 25% variance conditions. **(B)** Grand average of FIR event time courses extracted from the r/lCerebellum (0, -63, -24). FIR event time courses peak at about 20-s after block onset; **(C)** Grand average of Fitted event time courses extracted from the rCerebellum.

**FIGURE 12:**
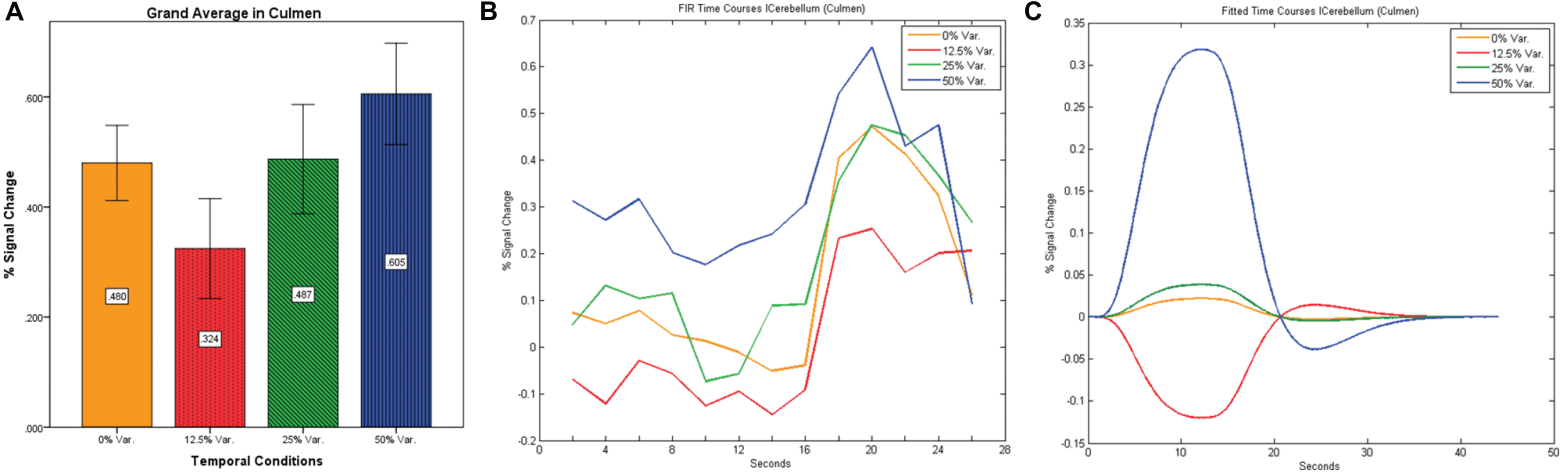
**(A)** Grand average of the mean percent signal change in lCulmen for all four conditions, showing a gradual increase in activation between conditions having variance in durations (i.e., 12.5, 25, and 50% variance) while the first condition with 0% variance showing a greater activation than the 12.5% variance conditions. **(B)** Grand average of FIR event time courses extracted from the lCulmen (-3, -57, -3). FIR event time courses peak at about 20-s after block onset; **(C)** Grand average of Fitted event time courses extracted from the lCulmen.

After identifying common ROIs based on global effects of a group analysis we extracted the time courses for each experimental condition from each ROI for each subject (with one exception – we did not include the Parahippocampal Gyrus in further analysis as being a direct result of the type of stimuli, i.e., Houses). Averaging the mean signal percent change of all subjects yielded the exact same pattern as in the FFA and PPA, that is, a gradual increase in activation between the 12.5, 25, and 50% variance conditions, while the 0% condition yielded a higher activation the 12.5% and the 25% variance conditions (see **Figures 9–12** showing mean percent signal change, FIR time courses and Fitted time courses for the right hemisphere ROIs). A one-way ANOVA test and a linear trend test using contrast coefficients of (-3, -1, 1, 3) over all four conditions, revealed a significant main effect as well as a significant linear trend, for all ROIs (see **Table 2**).

**Table 2:** One-way ANOVA and linear trend analysis.

MNI coordinates (x, y, z)	Talairach mapping	One-way ANOVA	Linear trend test Contrast coefficients: (-3, -1, 1, 3)
(3, 9, 6)	Right Caudate Body/Head	*F*(3,176) = 20.14, *p* < 0.0001	*t*(176) = 5.92, *p* < 0.0001
(-3, 15, 6)	Left Caudate Body/Head	*F*(3,176) = 19.55, *p* < 0.0001	*t*(176) = 5.48, *p* < 0.0001
(24, -33, 18)	Right Caudate Tail	*F*(3,176) = 28.14, *p* < 0.0001	*t*(176) = 5.70, *p* < 0.0001
(-6, 0, 12)	Left Caudate Body	*F*(3,176) = 13.11, *p* < 0.0001	*t*(176) = 4.56, *p* < 0.0001
(12, -15, 18)	Right Thalamus	*F*(3,176) = 13.96, *p* < 0.0001	*t*(176) = 4.59, *p* < 0.0001
(-9, -24, 12)	Left Thalamus – Pulvinar	*F*(3,176) = 17.42, *p* < 0.0001	*t*(176) = 5.61, *p* < 0.0001
(0, -63, -24)	Left/Right Cerebellum – Pyramis	*F*(3,176) = 18.11, *p* < 0.0001	*t*(176) = 2.99, *p* = 0.003
(-3, -63, -36)	Left Cerebellum – Inferior Semi-Lunar Lobule	*F*(3,176) = 20.12, *p* < 0.0001	*t*(176) = 3.15, *p* = 0.002
(-3, -57, -3)	Left Cerebellum – Culmen	*F*(3,176) = 6.81, *p* = 0.0002	*t*(176) = 2.73, *p* = 0.007

## Discussion and Conclusion

Our main finding in this study was the apparent representation of time in object category selective areas. As results indicate, category selective areas that are typically associated with the representation of shapes (i.e., faces or houses) are also sensitive to the variance in the exposure duration of these shapes. Consequently we conclude that temporal encoding is an integral part of perception.

Moreover, it appears that this sensitivity to variations in the durations of the stimuli does not appear in occipital (dorsal) regions such as the OFA and TOS, and thus should be assigned specifically to ventral regions (i.e., FFA and PPA). This conclusion also suggests that the effect found is not a general attention effect, or a global effect to variance, but rather reflects the specific sensitivity of these regions to variance in durations.

Our second finding relates to global effects associated with our temporal manipulation. Findings suggest that when disregarding stimuli specificity (faces vs. houses) and testing globally only for effects based on the amount of variance in duration, we find four distinct areas that seem to be sensitive to time (or at least to the duration variance of stimuli): (1) The Parahippocampal Gyrus; (2) the Thalamus; (3) the Basal Ganglia with specificity to the Caudate; and (4) the Cerebellum having three inner distinct sub-regions, the Culmen among them. While the involvement of the Parahippocampal Gyrus is directly related to the nature to the experiment, being sensitive to the specific stimuli presented, the involvements of the Thalamus, Caudate, and Cerebellum were less predictable.

A conclusion that can be drawn from the fact that our subjects were not informed of the temporal nature of the experiment and were engaged in a non-temporal task is that the sensitivity to duration variance is based on automatic processes which do not require attention or dedicated cognitive resources to process durations.

As results show, either locally within category selective regions or globally in the Cerebellum, Caudate, and Thalamus, the neural activation pattern was the same. While we expected an inclining gradient where the 0% variance condition will yield the minimum amount of activation while the 50% variance condition will yield the maximum neural activation, we found that this inclining gradient appears only within conditions where variance exist (i.e., 12.5, 25, and 50% variance) while 0% conditions yielded on average, a higher activation than the 12.5% and in several cases than the 25% variance conditions. We suspect that the higher activation of the 0% variance condition may lay on the fact that ecologically it differed from the other conditions as being the sole condition with no variance and may resemble the comparison of responses to shades of the red color (i.e., variance conditions) vs. the responses to the color blue (i.e., 0% condition).

### Implications on Duration Encoding and Time Perception Model

The present experiment contributes to the study of intrinsic and dedicated models of temporal representation. Results in this experiment suggest that on the one hand when looking for local representation of time, one should look within category selective brain regions that are typically associated with the encoding or representation of the specific type of stimulus at hand; while on the other hand, simultaneously, time is also represented globally. Based on the paradigm used in this study, this dual representation is not related to a temporal task or attentional resources allocated to temporal features.

As far as we know, this study is the first to report on temporal representation within the FFA and PPA with stimuli specificity. However, with respect to the Thalamus, Cerebellum, and the Caudate, this study seems to be in line with previous findings with two main exceptions: the first is that in the preset study no temporal or motor task were involved; and the second is that we used natural visual images of faces and houses stimuli, and thus the involvement of the Cerebellum, the Basal-Ganglia, and Thalamus in processing, or at least in being sensitive to variance in one of the visual stimuli properties (i.e., duration), was unexpected. The Cerebellum and the Basal Ganglia are common regions found to be associated with time processing. The Thalamus, however, is less common. Meck (1996) and Matell and Meck (2004) suggested that the Thalamus might be part of a time keeping circuit involving the Basal-Ganglia, which by itself is part of a larger time circuit involving the Cerebellum. Moreover, based on evidence showing high variability in time estimation in patients with lesions in Basal Ganglia and Thalamus, Gibbon et al. (1997) suggested that the Thalamus might have some sort of a regulatory function over information coming from the Cerebellum, or play a part in mediating information which is processed in the Basal Ganglia and transferred to the Putamen. Lee et al. (2007) suggested a more specific circuit of subseconds of time perception consisting of a Cerebral-Thalamus-Basal Ganglia-Cerebellum circuit. Several other studies report and suggest the involvement of the Thalamus in a loop network or circuit pertaining to time perception or time encoding (Ferrandez et al., 2003; Teki et al., 2011). The shared facet between most of these studies is that the Thalamus mediates information from the cortex to the inner ganglia (i.e., Basal Ganglia), or to deeper structures (i.e., the Cerebellum). In some models, the SMA and preSMA are also involved (Rao et al., 1997; Macar et al., 2004; Buhusi and Meck, 2005).

Our findings suggest that there should be a distinction in the way time is represented between the Cerebellum and the Thalamus on the one hand, and the Basal-Ganglia on the other. As can be seen in **Figure 10**, time courses in the Basal Ganglia peaks at about 10-s after block onset. This finding is in line with our findings of the way time courses in category selective areas behave under this specific manipulation. However, time courses in the Cerebellum and the Thalamus differ significantly from that pattern. As can be seen (see **Figures 9**, **11**, and **12**), time courses in these regions peak at about 20-s after block onset (during fixation time) suggesting a secondary role in temporal encoding that may be based on temporal information processed in the Basal-Ganglia. These findings are supported by Rao et al. (2001), which presents an Event-Related fMRI experiment also showing that temporal processing in the Basal-Ganglia occurs relatively early with respect to the Cerebellum which they assigned to the Cerebellum involvement in the process of timing rather than to the encoding of explicit timing.

Naturally, additional studies need to further investigate this issue, and better understand the nature of the delayed peak as well as the nature of the relationship between the Cerebellum, the Thalamus, and the Basal-Ganglia. Moreover, in order to generalize our findings relating to the representation or encoding of durations in category or feature selective regions, further exploration is needed.

## Conflict of Interest Statement

The authors declare that the research was conducted in the absence of any commercial or financial relationships that could be construed as a potential conflict of interest.

## Footnotes

1 http://www.fil.ion.ucl.ac.uk/spm/software/spm2/

2 http://marsbar.sourceforge.net/
